# Factors affecting the impact of covariate adjustment for binary outcomes in randomised clinical trials (RCTS): a simulation study

**DOI:** 10.1186/1745-6215-16-S2-P155

**Published:** 2015-11-16

**Authors:** Ly-Mee Yu, Douglas G Altman

**Affiliations:** 1 University of Oxford, Oxford, UK

## 

There is lack of consensus on whether analysis of RCT data should adjust for important baseline covariates. For binary outcomes, the estimated treatment effect can differ when logistic regression is carried out, even when the covariates are balanced between treatment groups. Simulation studies for RCTs with binary outcomes have shown that adjusting for a single baseline prognostic covariate increases the power to detect treatment effects. The impact on the size of the treatment effect was only notable when a strong prognostic covariate was included. The impact of adjustment for two covariates with different prognostic strengths and directions of the prognostic relationship to the outcome is not known. We present a detailed examination of factors affecting the impact of adjusted analysis. In particular, an adjusted logistic regression model with two covariates (1 continuous and 1 binary) are considered simultaneously. The simulation study addresses different levels of event rate and treatment effect, different distributions for the binary and continuous variables, variable prognostic strength of the covariates, and correlation between covariates. These simulations suggest that adjustment will only have a notable effect in extreme scenarios such as, treatment effect is very large and covariates are highly prognostic. We found that the relative difference between the unadjusted and adjusted odds ratios could be larger than 50%, though only under these scenarios. Otherwise the impact of adjustment is small.

